# An Economic Analysis of the Systematic Use of Mapping Systems during Catheter Ablation Procedures: Single Center Experience

**DOI:** 10.1155/2019/2427015

**Published:** 2019-08-20

**Authors:** Massimiliano Marini, Daniele Ravanelli, Marta Martin, Maurizio Del Greco, Fabrizio Guarracini, Silvia Quintarelli, Alessio Coser, Aldo Valentini, Roberto Bonmassari

**Affiliations:** ^1^Department of Cardiology, S. Chiara Hospital, Trento, Italy; ^2^Department of Physics, S. Chiara Hospital, Trento, Italy; ^3^Department of Cardiology, S. Maria del Carmine Hospital, Rovereto (TN), Italy

## Abstract

**Introduction:**

In this study we estimated the cost-effectiveness of adopting 3D Nonfluoroscopic Mapping Systems (NMSs) for catheter ablation (CA).

**Methods:**

This study includes patients who underwent supraventricular tachycardia (SVT) CA and atrial fibrillation (AF) CA from 2007 to 2016. A comparison was conducted between a reference year (2007) and the respective years for the two types of procedure in which the maximum optimization of patients' exposure using NMSs was obtained. We compared the data of all SVT CA performed solely using fluoroscopy in 2007 (Group I) and all SVT CA procedures performed using fluoroscopy together with an NMS in 2011 (Group II). There was also an important comparison made between AF CA procedures performed in 2007 (Group III) and AF CA in 2012 (Group IV), where patients' treatment in both years included the use of an NMS but where the software and hardware versions of the NMS were different. Two cost-effectiveness analyses were carried out. The first method was based on the alpha value (AV): the AV is a monetary reference value of avoided unit of exposure and is expressed as $/mansievert. The second one was based on the value of a statistical life (VSL): the VSL does not represent the cost value of a person's life, but the amount that a community would be willing to pay to reduce the risk of a person's death. The costs estimated from these two methods were compared to the real additional cost of using an NMS during that type of procedure in our EP Lab.

**Results:**

The use of NMS reduced the effective dose of about 2.3 mSv for SVT and 23.8 mSv for AF CA procedures. The use of NMS, applying directly AV or VSL values, was not cost-effective for SVT CA for the most countries, whereas the use of an NMS during an AF CA seemed to be cost-effective for most of them.

**Conclusions:**

In our analysis the cost-effectiveness of the systematic use of NMSs strongly depended on the AV and VSL values considered. Nonetheless, the approach seemed to be cost-effective only during AF CA procedures.

## 1. Introduction

At the present time, the first-line of therapy for many cardiac arrhythmias is considered to be radiofrequency catheter ablation (CA) [1, 2]. The procedure is regarded by many electrophysiologists as a safe and an effective procedure with a high acute success rate; however, it may require an extensive use of X-ray for catheter placement [3, 4]. The increased risk of developing cancer as a result of prolonged X-ray exposure time is well documented [5].

In the last fifteen years 3D nonfluoroscopic mapping systems (NMSs) have become established as an important tool for CA of complex arrhythmias. Studies published in research literature have, in addition, demonstrated that NMSs permit a significant reduction in X-ray exposure during standard CA procedures [6–10]. Nevertheless, daily use of NMSs during standard CA procedures is not a common practice in many EP Labs and the cost-effectiveness of using them remains an unknown.

Even with the reality of common practice in this area, the International Commission on Radiation Protection (ICRP) advises the adoption of the As Low As Reasonably Achievable (ALARA) concept in relation to the basic approach to the use of fluoroscopy in medicine [11].

### 1.1. Objectives

In an earlier paper, we have described that an extensive and daily use of NMSs in our context can drastically reduce X-ray exposure during CA procedures [12]. In this paper we carried out an economic analysis of this systematic approach in our EP Lab.

## 2. Methods

### 2.1. Study Population

This study is a retrospective analysis that involves consecutive patients who underwent supraventricular tachycardia (SVT) CA and atrial fibrillation (AF) CA procedures over 10 years of activity (from 2007 to 2016).

In our previous paper, we reported the fluoroscopic data of these procedures focusing the analysis on the reduction of radiation exposure and we have shown that the decrease in X-ray exposure year after year was due to the systematic use of NMSs rather than other variables. This reduction of exposure has occurred without compromising efficacy, safety, and long-term positive outcomes of CA [12].

In this study a comparison was conducted between a reference year (2007) and the respective years for the two types of procedure in which the maximum optimization of patients' exposure using NMSs was obtained. We compared the data of all SVT CA performed solely using fluoroscopy in 2007 (Group I) and all SVT CA procedures performed using fluoroscopy together with an NMS (EnSite NavX™ St. Jude Medical, St Paul, MN, USA) in 2011 (Group II). There was also a second important comparison made between AF CA procedures performed in 2007 (Group III) and AF CA in 2012 (Group IV), where patients treatment in both years included the use of an NMS but where the software and hardware versions of the NMS were different (CARTO XP 2007 and CARTO3 2012, Biosence-Webster, Diamond Bar, CA, USA).

Prior to each procedure, we obtained the written informed consent of the patient. Baseline patient characteristics of the four groups are summarized in Table 1. The procedures were performed as described in our previous paper [12].

### 2.2. Fluoroscopy and Procedure Data

For this study, we compared fluoroscopy time and total X-ray exposure. Fluoroscopy time (FT) was defined as the cumulative duration of fluoroscopy during the entire procedure, whereas the patient radiation dose was assessed as the recorded dose-area product (DAP).

### 2.3. Statistical Analysis

To examine the normal distribution of continuous variables, Shapiro-Wilk normality tests were performed. The nonparametric pairwise Wilcoxon Rank Sum tests were performed with Benjamin-Hochberg correction for multiple testing when required. The tests were used to compare all continuous variables and to exam median values between each of the years and each the groups for the respective procedures. To examine the categorical variables, the comparison between years and groups for the respective procedures was performed by means of the* χ*^*2*^ test, while for the follow-up and long-term effects the Kaplan-Meier analysis of Disease-Free Survival and the Log-Rank Test were used. Statistical analyses were conducted by means of R software, version 3.4.3 [13]* (The R Foundation for Statistical Computing)*. A p value of P<0.05 was considered significant.

### 2.4. Cost-Effectiveness Analysis

With the ICRP recommendations in mind, the economic analysis of X-ray dose reduction in CA procedures performed using NMSs was done utilizing two different methods: the alpha-value (AV) based method and the value of a statistical life (VSL) based method.

The two methods have their origin in the idea of effective dose (ED). ED is a factor put forward by the ICRP and represents an indicator of the risk related to a “standard” adult person (average age, gender, and weight), exposed to radiation. The total radiation detriment from stochastic effects associated with X-ray exposure is proportionally related to this risk-parameter.

As we have indicated in our previous paper [12], in our EP Lab the CA procedures are usually performed by using an X-ray fluoroscopy C-arm X-ray system. This system is equipped with a dose-monitoring device that registers the amount of radiation delivered to the patient. This device provides a value defined as dose-area product (DAP), i.e., the product of the irradiated area and the corresponding air-kerma value at the same distance. Since its implementation, the ED, expressed in Sievert (Sv) or submultiples as millisievert (mSv), has been a useful tool for X-ray detriment comparison between diagnostic and therapeutic techniques for the average adult. Applying an appropriate conversion factor equal to 0.20 [mSv/Gy*∗*cm^2^], as proposed in the EHRA Consensus Document [14] for “standard” adults, an estimation of ED is obtained for each year and each type of CA procedure in our study.

*The AV based method* adopts the concept of the alpha-value (AV) parameter. AV represents a monetary reference value of avoided unit of exposure and can be expressed as € or $/mansievert ($/man-Sv). This method is in general use in the nuclear industry as regards, specifically, workers in the industry and then more widely the population exposed to radiation. However, it is still unclear how it can be appropriately applied to the medical field due to the different conditions and rates of exposure. A notable limitation of the AV based method is the range of various values that the AV can have in different countries in which it is applied. Unfortunately, there is no worldwide standard AV value in use at the moment. This is due to the different fields in which it is considered such as economics, energy production, insurance, and health and protection in the workplace. In the last updated survey (2012) of the Information System on Occupational Exposure (ISOE), the European Technical Centre has published the AV values of regulatory bodies in different countries. This is reported in Table 2 [15] (column entitled AV value in $(2014-USD)/mSv). In 2016 the United States Nuclear Regulatory Commission (NRC) suggests the dollar per person-rem conversion, defined as the AV and only expressed in other units for cost-benefit analyses associated with radiation exposure, with a value updated to $5.200 (2014-USD) per person-rem, i.e., 520 $/man-millisievert ($/man-mSv) as reported in Table 2 [considering that 1 rem is equal to 10 mSv and 1 $ is equal to 1 dollar of United States (USD)] [16]. Despite these limitations the advantage of this method is a fast and easy cost-utility analysis since the cost reduction related to radiation saved is obtained by simply multiplying the ED reduction (ΔE, measured in [mSv]) with AV value.

Hence adopting AV based method and defining the Cost-Reduction as the reduction of the detriment cost related to the radiation dose saved for a single procedure with the use of NMS the parameters can be estimated using equation (A):

(A) Cost-Reduction = ΔE*∗*AV,

where AV values are expressed in [$/mSv] instead of [$/man-mSv], since being related to a single patient exposure.

*The Value of a Statistical Life (VSL)* based method aims at overcoming some limitations of the AV based method. It is based on the concept of the willingness to pay for specific level of risk reduction. The VSL does not represent the cost value of a person's life, but the amount that a community would be willing to pay to reduce the risk of one's death. The major advantage, with respect to the AV method, is that the economic quantification is performed after risk estimation and allows considering the age and the context of the population exposed to radiation (i.e., medical exposure). The risk assessment can be done using the coefficients reported in the recent (2011) report of the United States Environmental Protection Agency (EPA) EPA 402-R-11-001 [17]. This document estimates cancer incidence risk and cancer mortality risk due to low doses of ionizing radiation for the U.S. population.

The coefficient used to convert ED to lifetime attributable risk of cancer mortality has been estimated on the basis of the risk models described in EPA 402-R-11-001 [17]. The EPA's cancer risk model is also adopted by NRC for the dollar per person-rem conversion factor reassessment [16].

The lifetime risk (LR) of cancer mortality is strongly related to the age and sex of exposed patients. Hence we assumed for SVT CA that LR has an average value of 4.5 %/Sv for adult males and females at the age of 45. Instead for AF CA we assumed that LR has an average value of 3.8 %/Sv for adult males and females at the age of 60.

The economic quantification of the risk reduction shows the same limitation as for the AV value, i.e., the widespread range of values that VSL can assume. The VSL estimates differ across countries and also between agencies within the same country. The Organization for Economic Co-operation and Development (OECD) published in 2011 the VSL values [18]. For OCED countries, the recommended VSL range is $1.45–4.35 million (2005-USD), with a base value of $2.9 million (2005-USD), whereas for EU-27 countries, the recommendation is between $1.75–5.25 million (2005-USD), with a base value of $ 3.5 million (2005-USD) (see Table 3, column entitled VSL values in millions $(2014-USD)).

Even in the same country, for example the United States, the Office of Management and Budget (OMB) concludes that the majority of the studies across regulatory agencies on VSL suggest a value ranging from $1 million to $10 million (2001-USD) per statistical life in 2001 [19], updated to $ 1.2 million and $12.2 million (2010-USD) in 2010 [20].

The NRC staff has reviewed the VSL values proposed by different federal agencies and inflated them using formulas and guidance provided by the agencies. The NRC staff estimations are presented in Table 3 [16].

Hence, for a single procedure and adopting the VSL based method, the Cost-Reduction can be estimated using equation (B):

(B) Cost-Reduction = LARR*∗*VSL= ΔE *∗*LR*∗*VSL

The LARR is the Lifetime Attributable Risk Reduction, obtained as product of lifetime risk (LR) of cancer incidence related to X-ray exposure and the ED reduction (ΔE).

In this paper the cost-effectiveness and the sensitivity analysis were also performed as previously described in our work [21].

The extra cost of using an NMS during every SVT procedure has been calculated as €2,500{$3,454 ($2014-USD)} and €150 ($207 2014-USD) for each AF CA procedure.

## 3. Results

### 3.1. Total Population

Our study considered a total number of 155 patients who underwent SVT and AF CA procedures. The baseline characteristics of the four groups are summarized in Table 1. There are neither significant differences in the case of the two SVT groups nor any differences regarding the two AF groups in terms of procedural time, procedural success, and disease-free survival. However, this is not the case for FT and DAP which both show a statistically significant reduction in median values in each of the types of procedure.

### 3.2. Fluoroscopy and Procedure Data

The fluoroscopy data of Group I (38 patients who underwent SVT CA in 2007) was compared with the data of Group II (32 patients who underwent SVT CA in 2011). In the same way the fluoroscopy data of Group III (35 patients who underwent AF CA in 2007) was compared with the data of Group IV (50 patients who underwent AF CA in 2012).

In our EP Lab the use of an NMS was associated with reduced FT in Group II {median value 1.2 min, (95% CI [0.1-2.5]) (P<0.05)} compared to Group I {median value 16.6 min, (95% CI [12.2-21.8])}. There was a complementary reduction of the total X-ray exposure (DAP) from 12.70 Gy*∗*cm^2^ (95% CI [9.750-29.60]) in Group I to 1.03 Gy*∗*cm^2^ in Group II (95% CI [0.106-2.92]) (P<0.05). Similarly, the median ED during SVT CA procedures decreased from 2.54 mSv to 0.21 mSv, resulting in ED reduction ΔE equal to 2.33 mSv.

**Figure 1:**
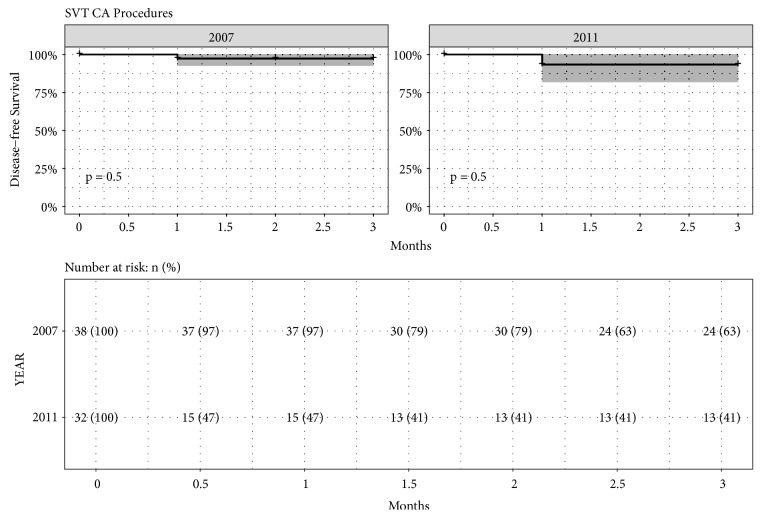
Kaplan-Meier estimates of disease-free survival with 95% confidence interval for SVT CA procedures for Group I (2007) and Group II (2011), with also the table of number of patients at risk reported as number and percentage (p value of Log-rank Test).

The reduction in X-ray exposure was obtained without prolonging the PT; there was a median value of 80 min (95% CI [70-108]) in Group I and a median value of 90 min (95% CI [72.5-106]) in Group II (P=NS). This occurred without any difference in the procedural outcome (P=NS) and achieved the same long-term effect in the follow-up (P=NS), as shown in Figure 1.

In the case of our EP Lab, the use of an NMS resulted in reduced FT in Group IV {median value 7.5 min, (95% CI [6.0-9.7]) (P<0.05)} compared to Group III {median value 49.1 min, (95% CI [41.4-56.6])}. There was a complementary reduction of the total X-ray exposure (DAP) from 138 Gy*∗*cm^2^ (95% CI [73.3-172]) in Group III to 18.9 Gy*∗*cm^2^ in Group IV (95% CI [14.8-23.4]) (P<0.05). Similarly, the median ED during AF CA procedures decreased from 27.6 mSv to 3.78 mSv resulting in a ED reduction (ΔE) equal to 23.82 mSv.

**Figure 2:**
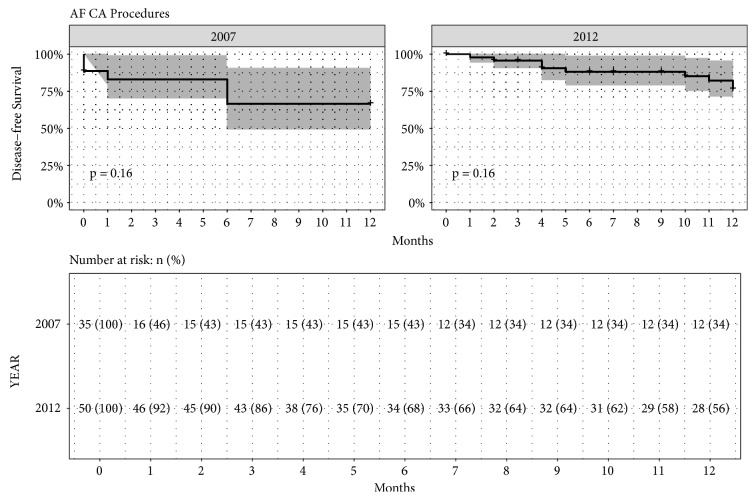
Kaplan-Meier estimates of disease-free survival with 95% confidence interval for AF CA procedures for Group III (2007) and Group IV (2012), with also the table of number of patients at risk reported as number and percentage (p value of Log-rank Test).

The reduction in X-ray exposure was achieved without prolonging the PT; there was a median value of 240 min (95% CI [220-240]) in Group III and a median value of 186 min (95% CI [154-200]) in Group IV (P=NS). This happened without any difference in the procedural outcome and attained the same long-term outcome in the follow-up (P=NS), as shown in Figure 2.

### 3.3. Economic Results

In this study we reported a reduction of 2.33 mSy using an NMS during SVT CA rather than SVT CA procedures performed with only fluoroscopy. Furthermore, we have also shown that the systematic use of an NMS, as well as the software and hardware updates of the NMS, has led to an ED reduction for each AF CA procedure equal to 23.82 mSv.

*Applying AV method* the cost-effectiveness of the CA procedures is strongly related to the AV values adopted in each country. In Table 2 we have reported the countries taken into account in this study. The reduction of the detriment is cost-related to the spared radiation dose in the use of an NMS during each type of CA procedure. The other two columns report if the use of an NMS is advantageous (Yes) or not (No), while the last two columns report the same results obtained using the higher and lower values of AV. For the majority of countries presented in Table 2 the dose reduction related to the use of NMSs during SVT CA is not advantageous with the exception of Switzerland. The economic threshold reference for SVT CA procedure in our EP Lab is $3,454 and for AF CA procedure is $207.

For example for the Netherlands, applying equations (A), (C), and (D) will result in the following.

(A) Cost-Reduction = ΔE *∗*AV = 2.33[mSv]*∗*619[$/mSv] =** $1,442.3** (2014-USD)

(C) High-Cost-Reduction = ΔE*∗*High AV = ΔE*∗*1.50*∗*AV = 2.33[mSv]*∗*1.50*∗*619[$/mSv] =** $2,163.4** (2014-USD)

(D) Low-Cost-Reduction = ΔE*∗*Low AV = ΔE*∗*0.50*∗*AV = 2.33[mSv]*∗*0.5*∗*619[$/mSv] =** $721.1** (2014-USD)

In contrast the systematic use of NMS during AF CA is economically advantageous for the majority of countries in Table 2.

For example for the Netherlands, applying equations (A), (C) and (D) will result in the following.

(A) Cost-Reduction = ΔE *∗*AV = 23.82[mSv]*∗*619[$/mSv] =** $14,744.58** (2014-USD)

(C) High-Cost-Reduction = ΔE*∗*High AV = ΔE*∗*1.50*∗*AV = 23.82[mSv]*∗*1.50*∗*619[$/mSv] =** $22,116.87** (2014-USD)

(D) Low-Cost-Reduction = ΔE*∗*Low AV = ΔE*∗*0.50*∗*AV = 23.82[mSv]*∗*0.5*∗*619[$/mSv] =** $7,372.29** (2014-USD)

*Applying the VSL method* the cost-effectiveness of the CA procedures is strongly related to the VSL values adopted in each country or organization and even in the same country; as for United States, there are different adopted VSL values across different agencies. In Table 3 we have reported the countries and agencies taken into account in this study with the reduction of the detriment cost related to the radiation dose sparing respectively with the use of NMSs during SVT CA and AF CA procedures. The other columns report if the use of NMSs is advantageous (Yes) or not (No), while the last two columns report the same results obtained using the higher and lower values of VSL.

Considering OECD published values, inflated and exchanged to $2014-USD as reported in Table 3, the economic advantageous of NMSs is not demonstrated for SVT CA.

For example for the VLS proposed by Food and Drug Administration (FDA), applying equations (B), (E), and (F) will result in the following.

(B) Cost-Reduction = LARR*∗*VLS = ΔE*∗*LR*∗*VLS = 2.33[mSv]*∗*4.5[%/Sv]*∗*8.6[million$] =** $901.7** (2014-USD)

(E) High-Cost-Reduction = LARR*∗*HighVLS = ΔE*∗*LR*∗*1,50VLS = 2.33[mSv]*∗*4.5[%/Sv]*∗*1.50*∗*8.6[million$] =** $1,353.6** (2014-USD)

(F) Low-Cost-Reduction = LARR*∗*LowVLS = ΔE*∗*LR*∗*0.50VLS = 2.33[mSv]*∗*4.5[%/Sv]*∗*0.50*∗*8.6[million$] =** $450.9** (2014-USD)

Again if we considered only the AF CA procedure, the use of NMSs seems to be cost-effective.

For example for the VLS proposed by Food and Drug Administration (FDA), applying equations (B), (E), and (F) will result in the following.

(B) Cost-Reduction = LARR*∗*VLS = ΔE*∗*LR*∗*VLS = 23.82[mSv]*∗*3.8[%/Sv]*∗*8.6[million$] =** $7,784.4** (2014-USD)

(E) High-Cost-Reduction = LARR*∗*HighVLS = ΔE*∗*LR*∗*1.50VLS = 23.82[mSv]*∗*3.8[%/Sv]*∗*1.50*∗*8.6[million$] =** 11,676.6$** (2014-USD)

(F) Low-Cost-Reduction = LARR*∗*LowVLS = ΔE*∗*LR*∗*0.50VLS = 23.82[mSv]*∗*3.8[%/Sv]*∗*0.50*∗*8.6[million$] =** 3,892.2$** (2014-USD)

## 4. Discussion

Nowadays the efficacy of the use of NMSs for the treatment of complex and simple arrhythmias has been well-demonstrated [22, 23]. An unexpected effect of this use is a significant reduction in fluoroscopy exposure and this advantage was validated in several papers based on randomized [9, 24, 25] and nonrandomized studies in adults [10, 26, 27] and paediatric populations [28]. However, regular use of an NMS during CA procedures is not enough to achieve the reduction in X-ray exposure; in fact, in our opinion a change in the mindset of the operators is fundamental in order to accomplish this reduction [12]. The use of simulators during EP training would be desirable.

In our previous paper we have analysed the cost-effectiveness of the use of NMSs during paediatric CA procedures and we noted the positive effect of this working method in combination with the new attitude, but only if we consider the correction factor for children and the higher values of AV and VLS [21]. This correction factor can be applied in children because they are more radiosensitive than adults.

In this study two cost-benefit methods (AV and VLS) have been introduced and applied to determine if the investment of a systematic use of NMSs during CA procedures in adults is economically advantageous in our EP Lab. During the analysis we have found the same problem as in the paediatric population of our previous study: the two methods show a very large range of parameters and this range of values is connected not only to the fact that there are different countries where they are used, but also to the fact that, even within the same country, different values are given by various agencies. Considering these limitations, the main conclusion of our study is that there is an important difference between SVT CA procedures and AF CA procedures. In SVT patients the effective dose reduction is very low and therefore economically disadvantageous considering the majority of AV and VSL values. This is in contrast to what is published in literature using a different economic analysis [9]. On the other hand, the effective dose reduction in AF patients is significant and, as a consequence, economically advantageous. Following from this, the major determinant for our economic analysis is the amount of ΔE. However, reducing the radiological exposure using a mapping system is not such an obvious thing, as it may seem in our experience. It is, in fact, the result of both a systematic use of NMS and a change in the electrophysiology team's mentality. Without this, a strong ED reduction in AF patients would not have been possible. In our economic analysis we did not take into account the positive effect of the X-ray reduction on the health of EP Lab workers and this is not a less important effect [5]. However, we have not collected worker doses and therefore further analysis is not possible.

### 4.1. Study Limitation

In the authors' opinion the limitations of this study can be summarized as follows:

1. The number of patients in this study is low.
2. The comparison between the third and fourth groups was not a comparison between AF CA procedures performed only with fluoroscopy and the same type of procedure performed with fluoroscopy plus an NMS. It can be assumed that without the use of the NMS the radiological exposure would have been greater in the third group, but this has not been demonstrated it in this study. We focused on comparison between the use of different software and hardware versions of NMS performing AF CA.
3. Since the CA procedure is a complex procedure the conversion from DAP to the effective dose was done using common and accepted methods of conversion, which resulted in what is, in fact, only an estimation of effective dose reduction.
4. Adopting the EPA's models for the quantification of cancer mortality risk, we did not consider the additional cost associated with not fatal cancers. Thus, the monetized benefit is underestimated.
5. The costs of NMSs usage are not standardized among institutions; thus the results could not be applicable to other centers.

## 5. Conclusions

Our study is a retrospective analysis and represents an economic evaluation of the systematic use of the NMSs during CA procedures in a real adult EP Lab context. The cost-effective advantageous of this use depends on which value of AV or VSL is applied. In our context the use of an NMS during SVT CA procedures results in being non-cost-effective considering the AV and VLS value of the most countries and agencies. Conversely, AF CA procedures with the use of a NMS seem to be cost-effective for most countries and agencies. This difference is related to the amount of dose reduction obtained during the procedure, which is very high during AF CA procedures. In our opinion this reduction is not only due to the use of NMS, but also due to the change in the mindset of the electrophysiologist. It is expected that the future decrease in unit cost of NMS will result in improved cost-effectiveness also for SVT CA procedures.

## Figures and Tables

**Table 1:** Clinical characteristics of the population of this study across groups.

		Group I SVT CA 2007	Group II SVT CA 2011	Group III AF CA 2007	Group IV AF CA 2012
		#	#	#	#
PROCEDURES		38	32	35	50

AGE	Mean [years]	47.0	45.1	56.9	60.7
	Standard deviation [years]	19.3	18.7	9.5	10.2

SEX	F	21	24	3	14
	M	17	8	32	36

HEART DISEASE	None	32	29	12	9
	Ischemic	1	0	2	3
	Dilated cardiomyopathy	1	0	2	3
	Hypertensive	3	7	13	28
	Valvular	1	0	4	1
	Congenital	0	0	0	0
	Other	0	1	2	6

LVEF	< 35%	1	0	0	1
	35-45%	0	0	2	1
	45-55%	0	0	5	4
	> 55%	37	32	28	40
	Not Available	0	0	0	4

Abbreviations: F, Female; M, Male; LVEF, Left Ventricular Ejection Fraction; AF, Atrial Fibrillation; SVT, Supraventricular Tachycardia; CA, Catheter Ablation.

**Table 2:** AV values, expressed as $(2014-USD)/mSv, in different countries, and corresponding Cost Reduction, expressed in $(2014-USD), Cost Effectiveness, expressed as Yes or No, Low and High Cost Effectiveness, both expressed as Yes or No, for SVT CA and AF CA, adopting AV method.

*Country [15]*	*AV-value in $(2014-USD)/mSv *	*SVT CA Cost Reduction $(2014-USD)*	*SVT CA Cost Effectiveness*	*SVT CA Low Cost Effectiveness*	*SVT CA High Cost Effectiveness*	*AF CA Cost Reduction $(2014-USD)*	*AF CA Cost Effectiveness*	*AF CA Low Cost Effectiveness*	*AF CA High Cost Effectiveness*
Canada	108	252	No	No	No	2573	Yes	Yes	Yes

Czech Republic (min value)	28	65	No	No	No	667	Yes	Yes	Yes

Czech Republic (max value)	137	320	No	No	No	3263	Yes	Yes	Yes

Finland	105	245	No	No	No	2501	Yes	Yes	Yes

Korea	84	196	No	No	No	2001	Yes	Yes	Yes

Netherlands	619	1445	No	No	No	14745	Yes	Yes	Yes

Romania	777	1814	No	No	No	18508	Yes	Yes	Yes

Slovakia	45	105	No	No	No	1072	Yes	Yes	Yes

Sweden (min value)	76	177	No	No	No	1810	Yes	Yes	Yes

Sweden (max value)	386	901	No	No	No	9195	Yes	Yes	Yes

Switzerland	3384	7898	Yes	Yes	Yes	80607	Yes	Yes	Yes

United Kingdom (min value)	17	40	No	No	No	405	Yes	Yes	Yes

United Kingdom (max value)	171	399	No	No	No	4073	Yes	Yes	Yes

United States	210	490	No	No	No	5002	Yes	Yes	Yes

*Country [16]*	*AV-value in $(2014-USD)/mSv *	*SVT CA Cost Reduction $(2014-USD)*	*SVT CA Cost Effectiveness*	*SVT CA Low Cost Effectiveness*	*SVT CA High Cost Effectiveness*	*AF CA Cost Reduction $(2014-USD)*	*AF CA Cost Effectiveness*	*AF CA Low Cost Effectiveness*	*AF CA High Cost Effectiveness*

United States	520	1214	No	No	No	12386	Yes	Yes	Yes

**Table 3:** VSL values, expressed as millions $(2014-USD), in different countries and United States Agencies, and corresponding Cost Reduction, expressed in $(2014-USD), Cost Effectiveness, expressed as Yes or No, Low and High Cost Effectiveness, both expressed as Yes or No, for SVT CA and AF CA, adopting VSL method.

*OECD [18]*	*VSL-values in millions $(2014-USD)*	*SVT CA Cost Reduction $(2014-USD)*	*SVT CA Cost Effectiveness*	*SVT CA Low Cost Effectiveness*	*SVT CA High Cost Effectiveness*	*AF CA Cost Reduction $(2014-USD)*	*AF CA Cost Effectiveness*	*AF CA Low Cost Effectiveness*	*AF CA High Cost Effectiveness*
OCED countries	3.5	369	No	No	No	3201	Yes	Yes	Yes
OCED EU-27	4.3	443	No	No	No	3842	Yes	Yes	Yes

*United States Agencies [16]*	*VSL-values in millions $(2014-USD)*	*SVT CA Cost Reduction $(2014-USD)*	*SVT CA Cost Effectiveness*	*SVT CA Low Cost Effectiveness*	*SVT CA High Cost Effectiveness*	*AF CA Cost Reduction $(2014-USD)*	*AF CA Cost Effectiveness*	*AF CA Low Cost Effectiveness*	*AF CA High Cost Effectiveness*

Environmental Protection Agency (EPA)	8.7	918	No	No	No	7958	Yes	Yes	Yes

Department of Transportation (DOT)	9.3	981	No	No	No	8507	Yes	Yes	Yes

Department of Homeland and Security (DHS)	8.6	907	No	No	No	7866	Yes	Yes	Yes

Food and Drug Administration (FDA)	8.6	907	No	No	No	7866	Yes	Yes	Yes

Occupational Safety and Health Administration (OSHA)	9.0	949	No	No	No	8232	Yes	Yes	Yes

Office of Management and Budget (OMB) (min value)	1.3	137	No	No	No	1189	Yes	Yes	Yes

Office of Management and Budget (OMB) (max value)	13.2	1393	No	No	No	12074	Yes	Yes	Yes

## Data Availability

The data used to support the findings of this study are available from the corresponding author upon request.
